# In-silico combinatorial design and pharmacophore modeling of potent antimalarial 4-anilinoquinolines utilizing QSAR and computed descriptors

**DOI:** 10.1186/s40064-015-1593-3

**Published:** 2015-12-29

**Authors:** Neha Parihar, Sisir Nandi

**Affiliations:** Division of Pharmaceutical Chemistry, Global Institute of Pharmaceutical Education and Research, Affiliated to Uttarakhand Technical University, Kashipur, 244713 India

**Keywords:** 4-Anilinoquinolines, Combinatorial library generation, Virtual screening, QSAR, Pharmacophore, Topological indices

## Abstract

There are very few studies for combinatorial library design and high throughput screening of 4-anilinoquinoline antimalarial compounds having activities against parasitic strain of *P. falciparum*. Therefore, an attempt has been made in the present paper to design potent lead compounds in this congener utilizing quantitative structure activity relationship utilizing theoretical molecular descriptors. QSAR models for a series of 4-anilinoquinolines considering various theoretical molecular descriptors including topological, constitutional, geometrical, functional group and atom-centered fragments has been carried out by stepwise forward–backward variable selections assimilating multiple linear regression (MLR) methods showing the topological indices contribute maximum impact on parasitic *P. falciparum* strain. A combinatorial library of 2160 compounds has been generated and finally, 16 compounds were screened through high throughput screening as promising 4-anilinoquinoline antimalarial hits based on their predicted activities utilizing topological descriptor based validated QSAR model. Highly predicted active compounds were then undergone for pharmacophore modeling to predict mode of binding and to optimize leads having greater affinity towards malarial *P. falciparum* parasitic strain.

**Electronic supplementary material:**

The online version of this article (doi:10.1186/s40064-015-1593-3) contains supplementary material, which is available to authorized users.

## Background

Malaria is an Anopheles mosquito borne parasitic disease triggered by four species of genus plasmodium including *P. falciparum*, *P. vivax*, *P. ovale*, and *P. malariae*. Amongst these, *P. falciparum* is the most dangerous species because it can penetrate into deeper tissues and infect red blood corpuscles leading to its breakdown and rupture, forming sticky lump like mass structure in the blood capillary which may ground circulatory arrest such as cerebral attack causing death of the individual (Tham and Kennedy 2015). As per the updated reports approximately 3.4 billion cases of malaria occur every year and about 1.3 million deaths occurred in the year of 2013 worldwide (Seder 2014). Brutal death of more than 1 million people globally cries to develop new antimalarial chemotherapeutics. One of the promising antimalarial chemotherapeutics is 4-anilinoquinoline derivatives including amodiaquine and piperaquine which act as blood schizontoside and haemazoin inhibitors. Due to drug resistance and lack of knowledge of exact mechanism of action of these series of compounds, it is really urgent to design and develop new congeneric leads utilizing structure activity-property relationship studies. Although the structure–property-activity relationships were developed since long years back (Crum-Brown and Fraser 1968), but now it is a multidisciplinary area of molecular design and are widely used for the prediction of properties, activities and/or toxicities of new chemicals by developing quantitative relationship between molecular activity or property (such as partition coefficient (log *P*), boiling point, melting point, acid and base constant, chromatographic retention index, toxicity, or reactivity) and computed structural properties such as constitutional, electrostatic, geometrical, topological, or quantum chemical molecular characteristics (Basak et al. 1997; Pompe and Novic 1999; Randic 1975; Roy et al. 2015a, b, c). Therefore in the present paper QSAR modelling has been carried out for antimalarial 4-anilinoquinolines based on the computed structure–property-activity correlations.

In this connection, a series of *N* ^1^,*N* ^1^-diethyl-*N*2-(4-quinolinyl)-1,2-ethanediamine derivatives having various groups substituted at the 7-position on the quinoline nucleus have been synthesized by Kaschula et al. (2002) who tested in vitro antimalarial activity of the same compounds against chloroquine sensitive D10 strain of *P. falciparum* showing that an electron attracting group at the 7th position bears with lower p*K*a of both the quinoline nitrogen atom and the tertiary anilino nitrogen in the alkyl side chain. O’Neill et al. (2003) synthesized a new series of amodiaquine analogues by interchanging hydroxyl at the 3′ position and the 4′ Mannich side-chain function of anilino moiety of quinoline which can produce non-toxic metabolite. Hwang et al. (2011) synthesized many 4-anilinoquinoline compounds introducing diaryl, ether, biaryl, and alkylaryl groups to the basic nucleus and tested their antimalarial activity against the chloroquine-sensitive strain 3D7 and the chloroquine-resistant K1 strain as well as for cytotoxicity against mammalian cell lines. In vitro screening and in vivo pharmacokinetic estimation of virtual libraries of newly designed chloroquine scaffold based 4-anilinonoquinolines showed highly potent antimalarial activity in mice found out two lead compounds utilizing ADMET predictions (Ray et al. 2010). Solomon et al. (2005, 2007) synthesized new 4-anilinoquinoline derivatives with evaluating its in vitro activity against chloroquine sensitive strain of *P. falciparum* strain and chloroquine resistant N-67 strain of *P. yoelii* in vivo whereas the same group generated another new series of 4-anilinoquinoline analogs which can form complex with hematin to act as hemazoin inhibitors showing affinity towards heme polymerization target.

To predict the biochemical mechanisms of 4-anilinoquinolines, quantitative structure–property-activity relationship studies were being executed recently by many researchers. Gupta et al. carried out QSAR on antimalarial activity and cytotoxicity of 4-anilinoquinoline using structural descriptors and identified that the antimalarial activity are being correlated with topological, 2D autocorrelation and functional group descriptors while cytotoxicity is being correlated with atom centered descriptors. This model suggests that the analogues with aromatic primary amines, aliphatic secondary amines are responsible for antimalarial activity and aromatic ethers, CH_2_R_2_ and CH_3_X contributed to cytotoxicity. With another work the author developed topological descriptor based QSAR model using electrons enrich species in aniline substituent showing better structure activity correlations quantitatively (Gupta et al. 2005; Gupta and Prabhakar 2006). Descriptors based QSAR modeling has been performed by many other authors and co-workers which are cited here (Masand et al. 2014; Sahu et al. 2014; Deshpande et al. 2009).

QSARs utilizing topological structural indices have been carried out but there is hardly any studies based on in silico virtual screening of combinatorial compounds and pharmacophore modeling of 4-anilinoquinoline compounds. One of the important techniques to focus mode of binding of the ligand is pharmacophore generation when the crystal structure of target is unknown. Delarue et al. synthesized a number of 4-anilinoquinolines having two proton accepting side chains and in vitro antimalarial activity has been evaluated on *P. falciparum* FcB1R strain whereas toxicity of the same compounds have been studied using MRC-5 cells and macrophages respectively (Delarue et al. 2001). A number of experimental and theoretical studies for the design of potent 4-anilinoquinolines have been performing. But experimental design of a single molecule involves a series of reactions and processes from the starting material of synthesis, structure elucidation and biological assays for activity studies. This total process consumes long years, enormous manpower, monetary issues and a number of animal sacrifices. So theoretical modeling utilizing QSAR based on topological indices computed solely from the structures of these compounds was carried out a lot. But there is scarcely any in silico design of 4-anilinoquinoline derivatives using pharmacophore modeling and virtual screening. In the present article, an attempt has been made to design thousands of combinatorial compounds at a time considering 4-anilinoquinoline scaffold with potential antimalarial activities. Such compounds are screened for potency and selectivity utilizing high-throughput screening techniques. HTS is based on lead optimization which incorporates Lipinski rule of five, QSAR and pharmacophore modeling. Such advances lead to greater understanding of new entity design having higher affinity towards the target.

## Methodology

### Experimental methods

#### Data base

A number of 4-anilinoquinolines having antimalarial activities against *P. falciparum* have been synthesized by Delarue et al. (2001). Different protons accepting side chains were substituted at 3′ and 5′ positions of the amino moiety to produce potent compounds which are tested for in vitro antimalarial activities against the chloroquine resistant *P. falciparum* FcB1R strain. Table 1 contains structure and antimalarial activities in terms of IC_50_ of 62 congeneric 4-anilinoquinoline derivatives. These IC_50_ values were converted into their negative logarithms (pIC_50_) which are taken into consideration in the present calculation as dependent variables whereas computed descriptors calculated by using optimized 3D-structure of 4-anilinoquinoline compounds are considered as independent variables for statistical multivariate regression modeling.

**Table 1:**
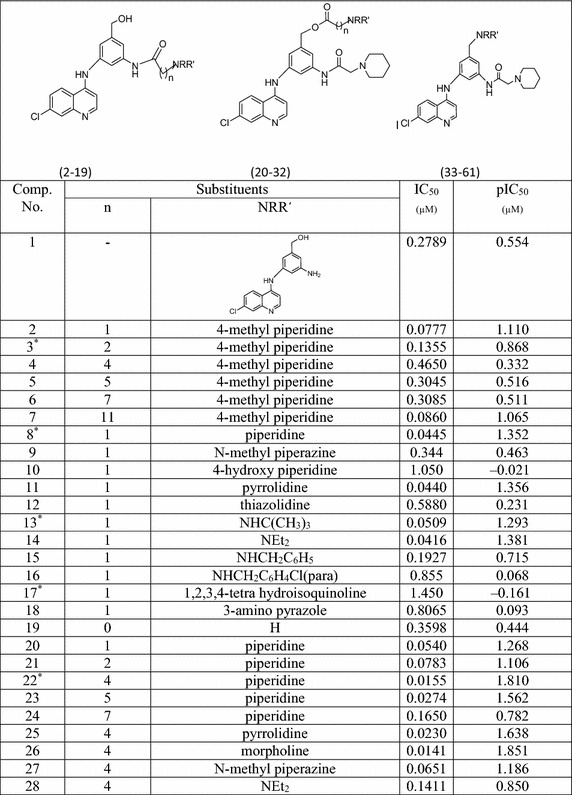

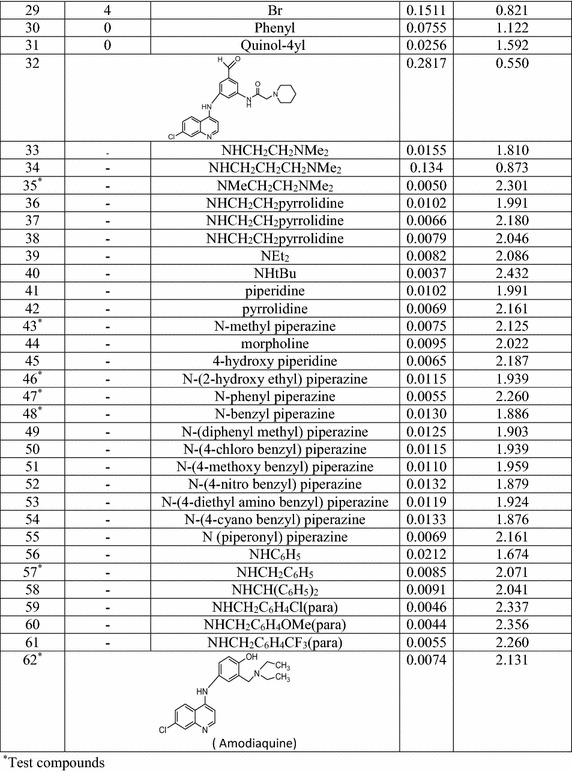
**Biological activity data**

Molecular optimization is carried out by minimization of molecular surface energy. For this purpose, 2D structures of 4-anilinoquinolines, drawn in ChemDraw software, were converted into 3D modules incorporated into Chem3D Ultra (Mills 2006). The 3D structures were energetically optimized using Merck Molecular Force Field with a value of 0.01 as Dielectric Constant.

Input MDL mol files of fully optimized molecules were then browsed into DRAGON software (Todeschini and Consonni et al. 2006, 2009) for computation theoretical structural descriptors. A total number of 1664 structural invariance including topological, 3D and geometrical, constitutional and molecular property, functional group and atom centered fragments have been calculated. The descriptors with same or almost near values or perfectly inter-correlated were reduced from the descriptor data to improve the degree of freedom. Thus, after reduction, a total number of 1367 different descriptors were selected for further quantitative-structure activity relationship modeling. Descriptor classes along with their names and standard symbols as calculated by the DRAGON software are given in Additional file 1: Table S1 (Batra et al. 2015).

### Statistical data analysis

The descriptor data has been analyzed by multiple linear regression (MLR) method. MLR can generate QSAR by correlating a set of computed structural invariance to compound’s antimalarial response endpoints. In the present data set sum of descriptors greatly beats the number of compounds. MLR may be applied when the numbers of descriptors are more or lower than the number of compounds (Batra et al. 2015; Katritzky et al. 2001; Tropsha et al. 2003; Draper and Smith 1998; http://www.minitab.com).

Since a large number of descriptor data have been calculated, so selection of variables is one of the decisive footsteps in QSAR modeling to predict the significant descriptors responsible for producing significant biological activities. If the association between the parameter(s) selected and activity is strong, then activity predictions will be possible. If there is only weak association, knowing the value of the parameter(s) will not help in predicting activity. Thus, for a given study, parameters should be selected which are relevant to the activity for the series of molecules under investigation and these parameters should have values which are obtained in a consistent manner. There are a number of methods for descriptor selection which includes genetic algorithm (de Campos and de Melo 2014; Broadhurst et al. 1997), stimulated annealing (Kirkpatrick et al. 1983), stepwise forward–backward selection (Hoskuldsson 1988; Nandi and Bagchi 2014), etc. Of them stepwise forward–backward feature selection is mostly user-friendly incorporated in Minitab software (http://www.minitab.com) which can select significant variables at 5 % level used in the present study for the generation of a number of QSAR models utilizing different sets of computed molecular descriptors including topological, 3D and geometrical, constitutional and molecular property, functional group and atom centered fragments, respectively.

F statistic value of 4.0 has been selected in the present calculation for inclusion and exclusion of the variables. Four different QSAR models have been formulated which were statistically validated by incorporating test and training sets approaches. The division of the total data set into training and test sets was performed at a random basis. Compounds with asterisk mark in Table 1 were selected as test set compounds. The quality of training model is denoted by R^2^ (R is the square root of multiple R-square for regression) and Q^2^ (cross-validation R^2^) respectively.

R^2^ and Q^2^ of a model are calculated by

$$ {\text{R}}^{ 2} = 1- \left[ {\sum \, \left( {{\text{Y}}_{\text{obs}} - {\text{Y}}_{\text{calc}} } \right)^{ 2} \Bigg/ \, \sum \left( {{\text{Y}}_{\text{obs}} {-}{\bar{\text{Y}}}} \right)^{ 2} } \right]\quad {\text{and}}\quad {\text{Q}}^{ 2} = { 1} - \left[ {\sum \, \left( {{\text{Y}}_{\text{obs}} {-}{\text{ Y}}_{\text{pred}} } \right)^{ 2} \Bigg/\sum \left( {{\text{Y}}_{\text{obs}} - {\bar{\text{Y}}}} \right)^{ 2} } \right] $$

where Y_obs_, Y_calc_ and Y_pred_ denote observed, calculated and predicted activity values, respectively, and ${\bar{\text{Y}}}$ indicates mean activity value of training molecules. Q^2^ denotes predictive statistics which should be greater than 0.5. The validated QSAR’s can identify the most significant contribution of the descriptor data modeled. Such most reliable validated model can be used to predict the highly active congeneric compounds which may be real or virtual, generated by combinatorial library design.

### Combinatorial library generation

Increase in the drug development cost and big pressure of discovering new molecules, pharmaceutical and biotech companies are crying to design new entity paying least money with increased profitability and productivity. The concept of combinatorial chemistry has been at the forefront of new molecule and drug discovery since 1990 but not quite as powerful a tool currently like a reliable pharmacophore model or structure-based methods (Ecker and Crooke 1995; Janda 1994; Davies 1996). However, one of the most efficient tools for design of millions of compounds paying least time and cost is computer aided combinatorial library generation. Incorporation of combinatorial library design and high throughput virtual screening including QSAR and pharmacophore modeling or structures based design as a major tools for lead optimization methods applied in the chemo-bioinformatics has dramatically altered the character of new lead discovery research paying least time and cost. Using traditional methods of synthesis, a medicinal chemist can produce limited number of compounds within certain time span. Early SAR studies are based on the use of physical properties and physicochemical substituent constants for the prediction of other more complex physicochemical, bio medicinal, and toxicological properties. Such property–property correlations are useful only when such properties for all compounds are available whereas on application of computer aided combinatorial library design, one can generate millions of compounds within a few time. Most of these compounds have no physicochemical data. Hence, there is a need to develop QSAR models using non-empirical parameters utilizing computed molecular descriptors for the screening of promising lead compound. Once high throughput screening started to make an impact the demand for optimized lead to test experimentally increased dramatically and the researchers began to develop new lead and scaffold more efficiently. The aim of this approach is to screen few potent lead like candidate structures which could be proposed for further synthesis, structure elucidation and biological activity testing using synthetic experiments (Lowe 1995; Terrett et al. 1995; Gallop et al. 1994; Nandi and Bagchi 2011a, b).

Generation of combinatorial chemical libraries is based on the designing of a scaffold which is a common substructure of the congeneric series. A number of different aliphatic and aromatic substituents are introduced at the specified substitution points of the common nucleus to produce large virtual libraries. In the present article, a total number of 2160 compounds have been generated by introducing different substituents at points of diversity including R_3_′, R_4_′, R_5_′ and R_7_, respectively associated to the parent 4-anilinoquinoline nucleus. The following Table 2 represents different possible substituents and the scaffold nucleus structures to develop combinatorial library.

**Table 2:**
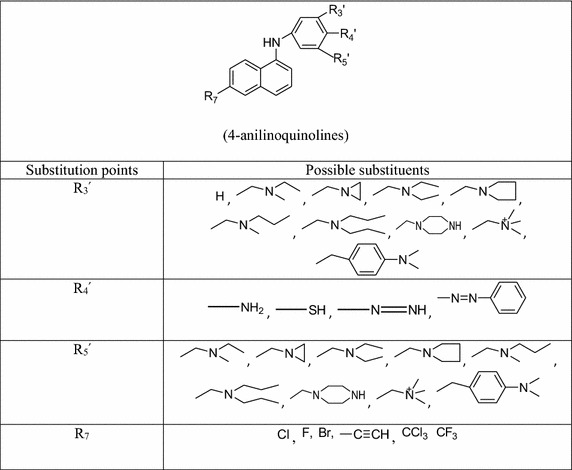
Scaffold and possible substituents attached to develop the virtual library

Virtual compounds were then screened by the application of high throughput screening techniques comprising of validated training QSAR, pharmacophore generation (Marshall et al. 1979; Beusen et al. 1999; Golender et al. 1993; Vilar and Koehlar 2000) and Lipinski’s ‘rule of five’ (Lipinski et al. 1997), respectively. The biological activities of the virtual compounds were predicted using the validated training QSAR model based on topological indices. Although this type of activity prediction is the conventional way for predicting active ligands, the method is not beyond contest as we do not have experimental measurement how so far the predicted activity is accurate. Therefore a comparative study between the observed activity of the known amodiaquine lead and predicted activity of the highly active virtual compounds and mode of binding prediction through pharmacophore modeling has been carried out for the highly predicted active congeneric compounds as well as active known leads (such as amodiaquine).

### Development of pharmacophore model

A pharmacophore consists of three-dimensional structural topographies for a given series of diverse molecules by ensuring the interaction of molecules with the biological target triggering the biological activity. It provides an estimate of common molecular interaction capabilities of a group of bioactive compounds for its target receptor structure. It does not represent any molecule or a functional group (Leach et al. 2009). All the active molecules sharing maximum number of common features are identified within the conformational flexible active binding region of space (Shoichet 2004; Mason et al. 2001). Therefore 3D pharmacophore assumes the mode of binding of structurally diverse molecules towards the biological target in a possibly common binding mode. These features are denoted as hydrogen bond donor, hydrogen bond acceptor, hydrophobicity of the moiety, aromatic rings, positive ionization properties (cation), negative ionization properties (anion), respectively (Langer and Krovar 2003; Koes and Camacho 2011). However, the concept is very insightful for understanding the molecular recognition aspects of a target receptor shared by a set of bioactive compounds. Pharmacophore modeling methods are not only useful for virtual screening and identification of new hits from databases but also useful for providing insights to de novo design of novel compounds and for understanding the complementary requirements for binding to the active sites of unknown candidate structures as well. Since pharmacophore transcends the chemical structural class and captures only the features responsible for activity, use of pharmacophore has the advantage for identification of potentially new biologically active compounds or chemical scaffolds as novel leads. Therefore in the present study an attempt has been made to focus on the 3D structural features based pharmacophore generation of 4-anilinoquinolines which are active against *P. falciparum* FcB1R using Portable InteLigandScout software (version 2.02) (Wobler and Langer 2005). Being a fully automated and convenient software tool, Ligand Scout is widely running on all operating systems with works being successfully published (Schuster and Langer 2005). In the present study common binding mode of the congeneric active ligands was analyzed by the development of pharmacophore model considering amodiaquine lead compound using default Ligand Scout settings. In addition to this pharmacophore model has been used as a predictive tool to optimize top 16 highly predicted active combinatorial compounds by generating its individual pharmacophore and compare with amodiaquine pharmacophore to correlate the mode of binding. Very interesting comparative predictive results were found which have been discussed in the next section.

## Results and discussion

### QSAR modeling

Earlier publications stated that topological indices can produce maximum impact on antimalarial activity of these congeneric compounds (Gupta 2015; Gupta and Prabhakar 2006; Masand et al. 2014; Sahu et al. 2014; Deshpande et al. 2009). Therefore in the present work a number of QSAR models were generated utilizing topological, functional group and atom centered fragments, constitutional and molecular property descriptors, respectively. Impact of different types of descriptors on antimalarial activities is focused in terms of R^2^ and its validation is done by calculating cross-validated R2 (R_cv_^2^) while treating the data set using MLR coupled with stepwise forward–backward selection methods. Outcomes were given in the following Table 3.

**Table 3:** Impact of descriptors on biological activity

Descriptor class	R^2^	R_cv_^2^
Topological	0.870	0.810
Functional group + atom centered fragments	0.812	0.744
Constitutional + molecular property	0.784	0.644
3d + geometrical	0.713	0.634

The above MLR models described that topological indices can produce highest influences in terms of R^2^ and R_cv_^2^ calculated as 0.870 and 0.810 followed by functional group and atom centered fragments, constitutional and molecular property, 3D and geometrical indices, respectively, which can contribute moderate impact on the inhibition of *P. falciparum* parasitic strain. Therefore, in the next attempt, topological indices have been selected to develop a number of QSAR models which are validated statistically by incorporating training and test sets concept as well as external validations. External validations are carried out by calculating predicted R^2^ and r_m_^2^ respectively. Topological descriptor based best training QSAR model, along with its quality and interpretation of modeled parameters are explained in the following Table 4. Predicted R^2^ and r_m_^2^ are calculated by the following formula.

$$ R_{\text{Pred}}^{2} = 1 - \frac{{\sum {\left( {Y_{{{\text{pred}}({\text{Test}})}} - Y_{{({\text{Test}})}} } \right)^{2} } }}{{\sum {\left( {Y_{(Test)} - \bar{Y}_{training} } \right)^{2} } }} $$

where, Y_pred(test)_ and Y_(test)_ indicate predicted and observed activity values respectively of the test set compounds and $\bar{Y}$_training_ indicates mean of observed activity values of the training set. For a predictive QSAR model, the value of R_pred_^2^ should be more than 0.5 (Nandi and Bagchi 2011a, b).

**Table 4:** Topological indices based training QSAR model and interpretation of the modeled descriptors

Validated training QSAR model	
$$ \begin{aligned} {\text{pIC5}}0 & = \left( { - 10. 2 3 4} \right) \, + \, \left( { - 9. 3} \right)*{\text{BIC5 }} + \, \left( { - 1. 8 3} \right)*{\text{GATS7e }} + \, \left( { - 3. 1 9} \right)*{\text{MATS7e }} + \, \left( { 4. 8 1} \right)*{\text{BEHe4 }} \\ & \quad + \, \left( { - 1. 2 1} \right)*{\text{EEig12r }} + \, \left( { - 0.0 6 5} \right)*{\text{DP12 }} + \, \left( { 1. 8 9} \right)*{\text{BELm7 }} + \, \left( { 3. 1} \right)*{\text{PCR}} \\ \end{aligned} $$ $$ \begin{aligned} {\text{N}} = 50,\, {\text{R}}^{ 2} = 0. 870, \quad {\text{Q}}^{ 2} = 0.810,\quad {\text{Pred}}\_{\text{R2 }} = 0. 7 3 7,\quad {\text{r}}_{\text{m}}^{ 2} = 0. 6 5 9, \hfill \\ {\text{Average r}}^{ 2}_{\text{m}} \left( {\overline{{{\text r}_{\text{m}}^{2} }} } \right) \, = \, 0. 6 8 2,\quad {\text{Delta r}}^{ 2}_{\text{m}} \left( \Delta {\text{r}}^{ 2_{\text{m}} } \right) = 0.0 4,\quad {\text{SEE}} = 0. 2 8 2 \end{aligned} $$	

Modeled descriptors	Interpretation
BIC5	Bond information content index (neighborhood symmetry of 5-order)
GATS7e	Geary autocorrelation—lag 7/weighted by atomic Sanderson electronegativities. 2D autocorrelations
MATS7e	Moran autocorrelation—lag 7/weighted by atomic Sanderson electronegativities
BEHe4	Highest eigen value of number 4 of burden matrix/weighted by atomic Sanderson electronegativities
EEig12r	Eigenvalue 12 from edge adj. matrix weighted by resonance integrals
DP12	Molecular profile number 12
BELm7	Lowest eigenvalue number 7 of Burden matrix/weighted by atomic masses
PCR	ratio of multiple path count over path count

Further, external predictability of the generated QSAR models was scrutinized by calculating modified r^2^ (r_m_^2^), average modified r^2^ ($\overline{{{\text{r}}_{\text{m}}^{2} }}$) and delta modified r^2^ (∆r_m_^2^), respectively which are given as

$$  {\text{r}}_{{\text{m}}}^{{\text{2}}}  = {\text{r}}^{2} \left( {1 - \left| {\sqrt {{\text{r}}^{2}  - {\text{r}}_{{\text{o}}}^{2} } } \right|} \right)  $$

where, r^2^ and r_o_^2^ are squared correlation coefficient between the observed (Y axis) and predicted (X axis) activity values of the test set with and without intercept, respectively. r_m_^2^ value must be greater than 0.5 to have a significant model (Roy and Roy 2008, 2009; Roy et al. 2013). Change of the axes gives the value of r′_0_^2^ and r′_m_^2^ is calculated by the following formula which depends on the value of r′_0_^2^.

$$ {{\rm r}^{\prime}}_{\text{m}}^{ 2} = {\text{r}}^{2} \times \left( {1 - \sqrt {{\text{r}}^{2} - {{\rm r}^{\prime}}_{ 0}^{ 2} } } \right) $$

where, r^2^ and r′_0_^2^ are squared correlation coefficient between the observed (X axis) and predicted (Y axis) activity values of the test set with and without intercept, respectively. Therefore, average r_m_^2^ and delta r_m_^2^ are now calculated by

$$ {\text{Average r}}^{ 2}_{\text{m}} \left( {\overline{{{\text{r}}_{\text{m}}^{2} }} } \right) = \left( {{\text{r}}^{ 2}_{\text{m}} + {{\rm r}^{\prime}}_{\text{m}}^{ 2} } \right)/ 2\quad {\text{and}}\quad {\text{delta r}}^{ 2}_{\text{m}} \left( {\Delta {\text{r}}^{ 2}_{\text{m}} } \right) \, = \, \left| {{\text{r}}_{\text{m}}^{ 2} - {{\rm r}^{\prime}}_{\text{m}}^{ 2} } \right| $$

It is noticeable that an acceptable QSAR model should give the value of “Average r_m_^2^” > 0.5 and “Delta r_m_^2^” should be <0.2, respectively. Values of modified r^2^ (r_m_^2^), average r_m_^2^ ($\overline{{{\text{r}}_{\text{m}}^{2} }}$) and delta r_m_^2^ (∆r_m_^2^) have been efficiently computed by web free software link of http://aptsoftware.co.in/ rmsquare/and http://203.200.173.43:8080/rmsquare/, respectively (Roy et al. 2013).

The selected model can explain and predict 87 and 81 % of variances of the antimalarial activity of the deliberated compounds. This model can also produce 73.7 % external predictability and r_m_^2^ value of 0.659 whereas values of average r_m_^2^ of 0.682 and delta r_m_^2^ of 0.04 extend more efficient evidence of external predictability of the generated QSAR model. Further the above QSAR model is confirmed its external predictability by predicting the response activities of the test molecules, as specified in Table 5.

**Table 5:** Predicted activity for the test set molecules

Compound number	Observed activity	Predicted activity
3	0.8681	0.754
8	1.3516	0.535
13	1.2933	1.362
17	−0.1614	0.153
22	1.8097	1.234
35	2.301	1.606
43	2.1249	1.909
46	1.9393	2.031
47	2.2596	2.051
48	1.886	2.013
57	2.0706	1.982
62	2.130	2.023

**Fig. 1:**
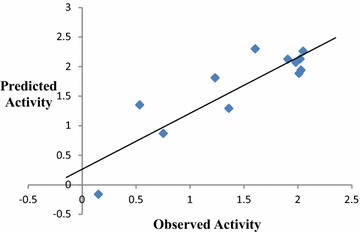
Observed versus predicted activities of the test molecules

From the Table 5 it is obvious that the predicted responses of all the test compounds are in good treaty with their corresponding observed responses and ideal fit is attained produced by plotting a graph (Fig. 1) by correlating observed activity versus predicted activity of the test set compounds. The squared correlation coefficient is calculated as 0.771.

Once the QSAR model formulated and validated properly, its utility is to predict the biological responses of the compounds which are generated by combinatorial deign and experimentally non-investigated.

### Combinatorial library generation and virtual screening

In the present study a total number of 2160 compounds have been generated by introducing a number of 10, 4, 9 and 6 different substituents at various substitution points including R_3_′, R_4_′, R_5_′ and R_7_′, respectively connected to the common template of 4-anilinoquinoline. The rationale behind this group selection is to undergo literature survey to find out the active lead in these congeners including amodiaquine and isoquine respectively (O’Neill et al. 2003). Let us consider the different functional groups associated to the scaffold of these lead compounds and modify these substituents based on the developed pharmacophore model for active lead. The special feature of the new family reported here are the presence of basic side chain at both the 3′ and 5′ positions and therefore the impossibility of nucleophillic addition of proteins even in the case of a metabolic hydroxylation at the hindered 4′-site (Delarue et al. 2001). R_4_′ position should be substituted by bio-isosteres of –OH group whereas electron drawing moiety is favorable at R_**7**_ position of the common substructure.

All the optimized virtual compounds were screened by predicting biological activities in terms of pIC_50_ utilizing best topological indices based training QSAR model described in Table 4. As per the prediction, a number of top 16 highly predicted active compounds (Table 6) were reported as hits for further lead optimization process. It was shown that predicted activity of all these 16 highly active virtual hits much greater than the AQ lead. It was also calculated that these highly predicted active virtual compounds match with the property ranges as prescribed by Lipinski’s ‘Rule of Five’ which include following properties such as number of hydrogen bond acceptor, number of hydrogen bond donor, XlogP, molecular weight and rotatable bond count respectively.

**Table 6:**
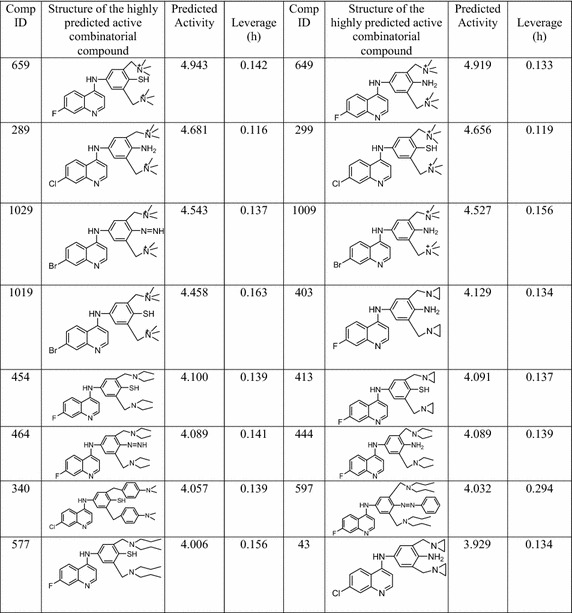
Top 16 highly predicted active compounds along with their predicted biological activity

As none of these virtual compounds are experimentally tested, it is very vital to test whether these compounds are within the chemical applicability domain (AD) of the developed model, especially in view of that all 16 hit molecules have biological activity values much higher than those of the training set compounds (i.e., the hit compounds are outside the activity domain of the training molecules). The applicability domain of a training QSAR model determines its acceptance by the regulatory bodies such as Organization for Economic Cooperation and Development (OECD) for its applications to predict new molecules. The OECD Principle 3 defines ‘a defined domain of applicability’ for the developed QSAR model. The Setubal Workshop report (Jaworska et al. 2005) presented the following regulation for the AD assessment: “The applicability domain of a (Q)SAR is the physico-chemical, structural, or biological space, knowledge or information on which the training set of the model has been developed, and for which it is applicable to make predictions for new compounds. The applicability domain of a (Q)SAR should be described in terms of the most relevant parameters, i.e., usually those that are descriptors of the model. Ideally the (Q)SAR should only be used to make predictions within that domain by interpolation not extrapolation” (OECD 2007).

In the present study, applicability domain of the training model as well as top 16 virtual 4-anilino quinolone hits were calculated by using “AD using Standardization approach” which is a free ware tool (Roy et al. 2015a, b, c) to find out whether query compounds are located outside the applicability domain of the built QSAR model and it also detects outliers present in the training set compounds. The results depicted that training molecules 1 and 19 were detected as outlier whereas all the predicted hits are situated within the zone of AD.

Further, to cross check the accuracy of this model validation, leverage value (h) and warning leverage (h*) for each of screened hits were calculated. The leverage value (h) of a compound in the original variable space which measures its influence on the model may be defined as

$$ {\text{h}}_{\text{i}} = {\text{ x}}_{\text{i}}^{\text{T}} \left( {{\text{X}}^{\text{T}} {\text{X}}} \right)^{ - 1} {\text{x }}; \quad \left( {{\text{i }} = { 1},{ 2}, \, \ldots ,{\text{ n}}} \right) $$

where, x_i_ is the descriptor row-vector of the i-th compound, x_i_^T^ is the transpose of x_i_, X is the descriptor matrix, X^T^ is the transpose of X (X^T^X)^−1^ is the inverse of matrix X^T^X.

$$ {\text{The warning leverage }}\left( {{\text{h*}}} \right){\text{ may be calculated by h*}} = 3 {\text{k}}/{\text{n}} $$

where, n is the number of training compounds and k is the number of model parameters (Hong et al. 2009; Hemmateenejad and Yazdani 2009; Nandi et al. 2011). The leverage value of all hit compounds was mentioned in Table 6. The calculated warning leverage value is of 0.480. A leverage (h) greater than warning leverage (h*) means that the predicted response is the result of substantial extrapolation of the model and therefore may not be reliable. For the present investigation, it was observed that the leverages of all hit compounds are lower than h* which are pretty acceptable.

### Further lead optimization through pharmacophore modeling

**Fig. 2:**
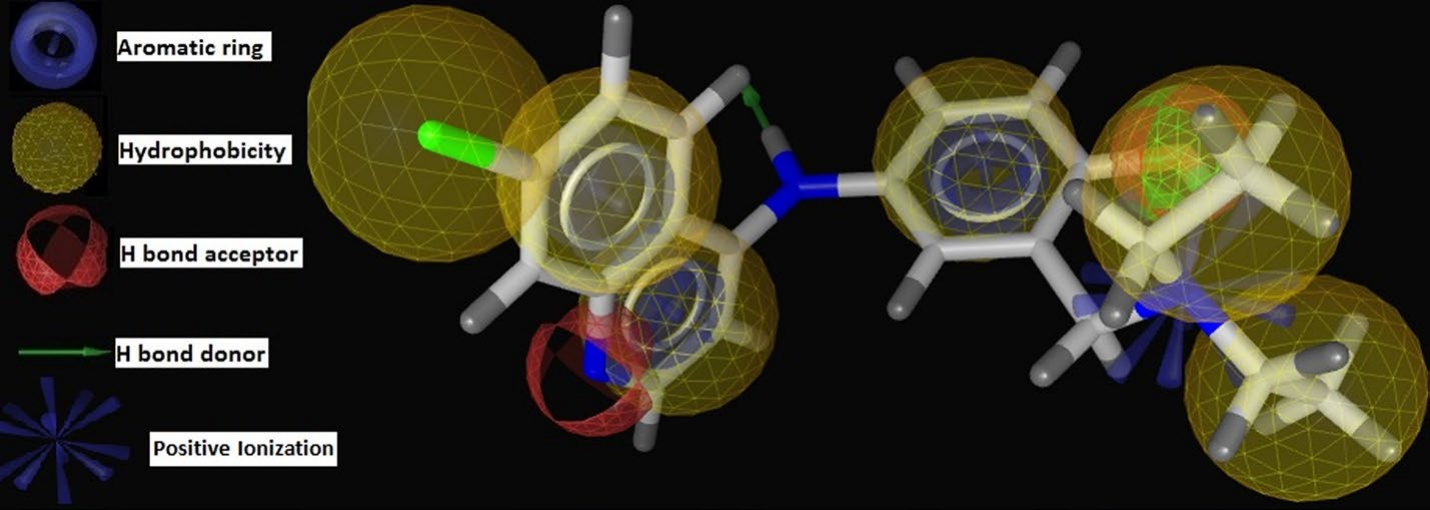
Pharmacophore of amodiaquine (AQ)

Finally, 16 compounds were predicted as promising 4-anilinoquinoline hits active against chloroquine resistant *P. falciparum* FcB1R strain. As the crystal structure of the *P. falciparum* target is unknown, therefore, the predicted hits were subjected to pharmacophore generation to investigate the mode of interaction with the receptor target. Fully optimized 3D structure of AQ was considered as a reference for pharmacophore generation because AQ is an established potential lead-like drug in this in 4-anilinoquinoline congeneric series. To focus the inhibitor’s crucial features required for binding with malarial *P. falciparum* FcB1R strain, a comparative study between the amodiaquine (lead) and 4-anilinoquinoline (highly active virtual compounds) pharmacophore has been studied. The pharmacophore model generated by us for amodiaquine (lead) is given in Fig. 2.

The above model predicted five features including three aromatic points (blue circle), six hydrophobicity (yellow ball), two HBAs (orange color), two HBDs (green arrow and lawn green ball) and one positive ionization (blue star). Quinoline nucleus itself should be aromatic and hydrophobic. N1 of the quinoline is a hydrogen bond acceptor whereas 4-amino group is hydrogen bond donor. 4-anilino benzene contributes aromaticity. R_3_′ may interact with the target by creating hydrophobicity and positive ionization. R_4_′ can produce hydrogen bond interaction. Electron withdrawing moiety is favorable at R_7_ position of the quinoline nucleus which is also responsible for producing hydrophobic interaction.

The detailed comparative pharmacophoric 3D features for AQ (lead) and top 16 predicted active congeners have been given in the following Table 7.

**Table 7:** Comparative pharmacophoric 3D features for AQ (lead) and top 16 predicted active congeners

Compound ID	Pharmacophoric 3D features predicted by our models							
	Quinoline	N1	4-amino	4-anilino benzene	R_7_	R′_3_	R′_4_	R′_5_
AQ (Lead)	H; AR	HBA	HBD	AR	H	PI; H	HBA; HBD	-
659	H; AR	HBA	HBD	AR	H; HBA	PI	HBA;HBD	PI
649	H; AR	HBA	HBD	AR	H; HBA	PI	HBD	PI
289	H; AR	HBA	HBD	AR	H	PI	HBD	PI
299	H; AR	HBA	HBD	AR	H	PI	HBA;HBD	PI
1029	H; AR	HBA	HBD	AR	H	PI	HBA;HBD	PI
1009	H; AR	HBA	HBD	AR	H	PI	HBD	PI
1019	H; AR	HBA	HBD	AR	H	PI	HBA;HBD	PI
403	H; AR	HBA	HBD	AR	H; HBA	PI	HBD	PI
454	H; AR	HBA	HBD	AR	H; HBA	PI; H; H	HBA;HBD	PI; H; H
413	H; AR	HBA	HBD	AR	H; HBA	PI	HBA;HBD	PI
464	H; AR	HBA	HBD	AR	H; HBA	PI; H; H	HBA;HBD	PI; H; H
444	H; AR	HBA	HBD	AR	H; HBA	PI; H; H	HBD	PI; H; H
340	H; AR	HBA	HBD	AR	H	AR; H; H	HBA;HBD	AR; H; H
597	H; AR	HBA	HBD	AR	H; HBA	PI; H; H	HBA; H; AR	PI; H; H
577	H; AR	HBA	HBD	AR	H; HBA	PI; H; H	HBA;HBD	PI; H; H
43	H; AR	HBA	HBD	AR	H	PI	HBD	PI

*H* hydrophobicity, *AR* aromaticity, *HBD* hydrogen bond donor, *HBA* hydrogen bond acceptor, *PI* positive ionization

For AQ lead and rest of the highly predicted active compounds considered by us after screening (Table 7), it is seen that the aromatic quinoline ring should interact with hydrophobic residues. N1 of the quinoline, 4-amino and R_4_′ of the aniline moiety may produce hydrogen bond interactions. 4-anilino benzene shows aromaticity. R_7_ position of the quinolone interacts with hydrophobic residues whereas R_3_′ and R_5_′ must be substituted by the groups which contribute positive ionizations responsible for ligand receptor interactions. Therefore this is an important attempt for prediction of biochemical mechanisms of the top virtual hits generated by combinatorial library design. Although experimental validation of the screened hits are necessary utilizing in vitro and in vivo analyses, however, an integration of pharmacophore modeling, virtual screening, structure-based methods, molecular biology and combinatorial chemistry together can provide a better basis for more efficient drug discovery and design reducing both costs and time.

### New focus on compound’s mechanism of action

**Fig. 3:**
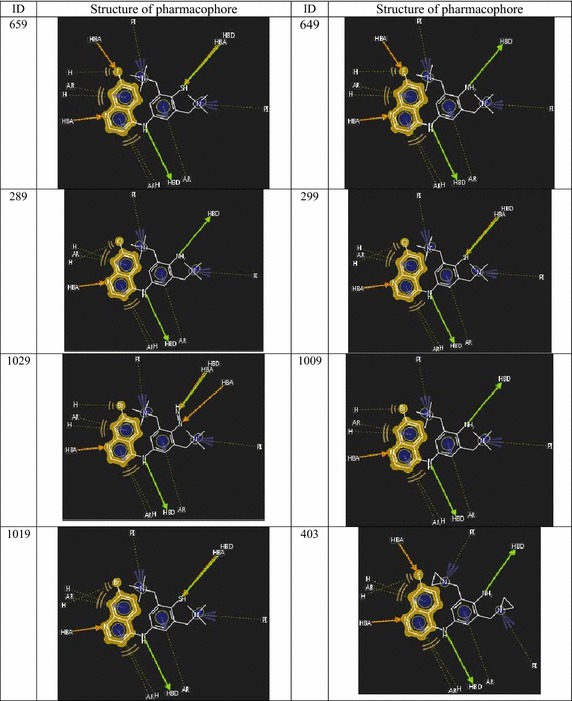
Pharmacophore models of selected highly active virtual hits

**Fig. 4:**
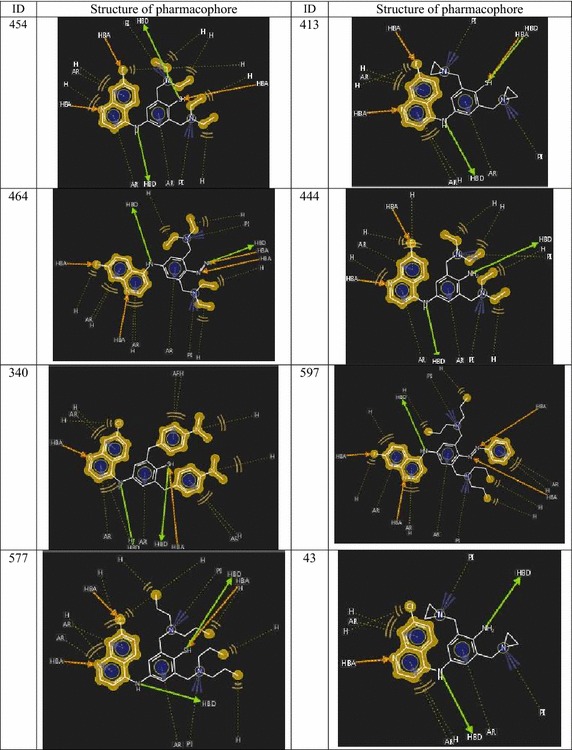
Pharmacophore models of selected highly active virtual hits

When the pharmacophore models developed for screened highly active combinatorial hits (Figs. 3, 4) were compared with the AQ lead, a significant comparable performance was noted in terms of mode of interactions with the target protein. As per the prediction, compound ID 659 and 649 were predicted as highest active hits with predicted activities (pIC50) are 4.943 and 4.919 µM respectively. The pharmacophoric interaction patterns of selected active hits were shown in Figs. 3 and 4. Some more active virtual hits were predicted as ID 454, 464, 444, 597 and 577 of those modes of binding and predicted activity are likely with compound ID 659 and 649.

The predicted activities of these two compounds and mode of bindings are almost similar, there is a sharp change in pharmacophore when compared with AQ lead and the other two compounds. From the pharmacophore models (Figs. 3, 4) it is clear that R_3_′ and R_5_′ must be substituted by the basic groups containing tertiary amino moiety responsible for producing positive ionization (PI). More PI in these regions can increase affinity of the ligand towards negative environment of the acidic parasitic digestive vacuoles. R_7_ electron withdrawing moiety of quinoline may produce hydrogen bond interaction with the target protein. These added pharmacophoric features in compare with AQ (lead) pharmacophore may enhance the antimalarial activity of these compounds. The special feature of the new family already reported is the presence of a basic side chain at both the R_3_′ and R_5_′ positions and therefore the impossibility of nucleophillic addition of proteins even in the case of a metabolic hydroxylation at the hindered R_4_′ site (Delarue et al. 2001). Metabolic hydroxylation of R_4_′ substituent may produce toxic metabolite (O’Neill et al. 2003). To generate least toxic and highly active compounds an attempt has been made in the current study for the designing of 4-anilinoquinoline compounds by substituting thiol, diazo, phenyl diazo and amino groups instead of hydroxyl group.

## Conclusion and future direction

The advance research, so far yet, focused that 4-anilinoquinolines are basic in nature. They can deposit into the acidic digestive vacuoles of the plasmodium and interact with the heme and interfere with the parasitic DNA sequestration (Valderramos and Fidock 2006). From the present study of pharmacophore modeling, it has been found that tertiary amino group (basic) associated with R_3_′ and R_5_′ positions impart positive ionizations. Parasitic nucleic acid bases such as quinine and uracil may undergo nucleophillic attack. The tert-N-group of the compound may interact with this guanine and uracil bases via positive ionization and thus breaks the DNA chain length of the malarial parasite. R_3_′ and R_5_′ substituents such as dialkylaminoalkyl moiety may also impart hydrophobicity and cause hydrophobic interaction with the hydrophobic amino acid residues such as histidine of the parasitic proteins. Heme is bound with histidine and lipid to undergo DNA sequestration. Thus positive charge ionization and hydrophobicity are responsible to inhibit DNA sequestration. Therefore the virtual compounds ID including 454, 464, 444, 597 and 577 are predicted as highly active hits in this congeneric series. Compounds ID 659 and 649 are predicted as highest top two active lead like compounds because R_7_ electron withdrawing moiety of these compounds may contribute an additional hydrogen bond interaction which is decisive for producing antimalarial activity. In comparison to AQ lead, other predicted active hits ID including 289, 299, 1029, 1009, 1019, 340 and 43 bear almost same mode of pharmacophoric interaction patterns with an additional feature of PI at R_5_′ position. Therefore, these predicted active virtual compounds may be recommended for further synthesis and testing as potent agents against *P. falciparum* FcB1R strain. Studies in this direction may help to design new congeneric active leads with least toxicity. Further, synthesis, testing for activity and toxicity study may be carried out in near future to model potent antimalarial compounds in this series.

## Additional file

**Additional file 1: Table S1.** Computed theoretical molecular descriptors used in the study.
