# Supplementary material for: In-silico combinatorial design and pharmacophore modeling of potent antimalarial 4-anilinoquinolines utilizing QSAR and computed descriptors
Source: Springerplus. 2015 Dec 29;4(1):819. doi: 10.1186/s40064-015-1593-3 (PMC5590512; doi:10.1186/s40064-015-1593-3)
Supplement: Supplementary file 1 — Additional file 1: Table S1. Computed theoretical molecular descriptors used in the study. [file 40064_2015_1593_MOESM1_ESM.docx]

**Supplementary Material**

^†^**Table S1**: Computed theoretical molecular descriptors used in the study.

| Descriptor Classes | Examples |
| --- | --- |
| .  Geometrical  and  Three Dimensional (3D) | Gravitational index G1 (G1), gravitational index G2 (bond-restricted) (G2), radius of gyration (mass weighted) (RGyr), (W3D), (J3D), (H3D), (AGDD), (DDI), (ADDD), span R (SPAN), average span R (SPAM), molecular eccentricity (MEcc), spherosity(SPH), asphericity(ASP), Folding degree index (FDI), 3D Petitjeanshape index (PJI3), length-to-breadth ratio by WHIM (L/Bw), (SEig), Harmonic Oscillator Model ofAromaticity index (HOMA), ring complexity index (RCI), aromaticity index (AROM), HOMA total (HOMT), displacementvalue / weighted by mass (DISPm), quadrupole x-component value / weighted by mass (QXXm), quadrupole y-componentvalue / weighted by mass (QYYm), quadrupole z-component value / weighted by mass (QZZm), displacement value /weighted by van der Waals volume (DISPv), quadrupole x-component value / weighted by van der Waals volume (QXXv), quadrupole y-component value / weighted by van der Waals volume (QYYv), quadrupole z-component value / weightedby van der Waals volume (QZZv), displacement value / weighted by Sanderson electronegativity (DISPe), quadrupolexcomponentvalue / weighted by Sanderson electronegativity (QXXe), quadrupole y-component value / weighted bySanderson electronegativity (QYYe), quadrupole z-component value / weighted by Sanderson electronegativity (QZZe), displacement value / weighted by polarizability(DISPp), quadrupole x-component value / weighted bypolarizability(QXXp), quadrupole y-component value / weighted by polarizability(QYYp), quadrupole z-component value / weighted by polarizability(QZZp), sum of geometrical distances between N..N(G(N..N)), sum of geometrical distancesbetweenN..O(G(N..O)), sum of geometrical distances between N..S(G(N..S)), sum of geometrical distances betweenN..F(G(N..F)), sum of geometrical distances between N..Cl(G(N..Cl)), sum of geometrical distances between N..Br(G(N..Br)), sum of geometrical distances between O..O(G(O..O)), sum of geometrical distances between O..S(G(O..S)), sum of geometrical distances between O..F(G(O..F)), sum of geometrical distances between O..Cl(G(O..Cl)), sum of geometrical distances between O..Br(G(O..Br)), sum of geometrical distances between S..Cl(G(S..Cl)), sum of geometrical distances between F..Cl(G(F..Cl)), sum of geometrical distances between Cl..Cl(G(Cl..Cl)), sum of geometrical distances between Cl..Br(G(Cl..Br)), (ITH), (ISH), (HIC), (HGM), (H1u), (H2u), (H3u), (H4u), (H5u), (H6u), (H7u),(H8u), (HTu), (HATS0u), (HATS1u), (HATS2u), (HATS3u), (HATS 4u), (HATS5u), (HATS6u), (HATS7u), (HATS8u), (H0m), (H1m), (H2m), (H3m), (H4m), (H5m), (H6m), (H7m), (H8m), (HTm), (HATS0m), (HATS1m), (HATS2m), (HATS3m), (HATS4m), (HATS5m), (HATS6m), (HATS7m), (HATS8m), (HATSm), (H0v), (H1v), (H2v), (H3v), (H4v), (H5v) (H6v), (H7v), (H8v), (HTv) (HATS0v), (HATS1v), (HATS2v), (HATS3v), (HATS4v),(HATS5v), (HATS6v), (HATS7v), (HATS8v), (HATSv), (H0e), (H1e),H2e), (H3e), (H4e), (H5e), (H6e), (H7e),(H8e),(HTe), (HATS0e), (HATS1e), (HATS2e), (HATS3e), (HATS4e), (HATS5e), (HATS6e), (HATS7e), (HATS8e), (HATSe), (H0p), (H1p), (H2p), (H3p), (H4p), (H5p), (H6p), (H7p), (H8p), (HTp), (HATS0p), (HATS1p), (HATS2p), (HATS3p), (HATS4p), (HATS5p), (HATS6p), (HATS7p), (HATS8p), (HATSp), (RCON), (RARS), (REIG), (R1u), (R2u), (R3u), (R4u), (R5u), (R6u), (R7u), (R8u), (RTu), (R1u+), (R2u+), (R3u+), (R4u+), (R5u+), (R6u+), (R7u+), (RTu+), (R1m), (R2m), (R3m), (R4m), (R5m), (R6m), (R7m), (R8m), (RTm), (R1m+), (R2m+), (R3m+), (R4m+), (R5m+), (R6m+), (R7m+), (R8m+), (RTm+), (R1v), (R2v), (R3v), (R4v), (R5v), (R6v), (R7v), ((R8v), (RTv), (R1v+), (R2v+), (RTv+), (R1e), (R2e), (R3e), (R4e), (R5e), (R6e), (R7e), (R8e), (RTe), (R1e+), (R2e+), (R3e+), (R4e+), (R5e+), (R6e+), (R7e+), (R8e+), (RTe+), (R1p), (R2p), (R3p), (R4p), (R5p), (R6p), (R7p), (R8p), (RTp), (R1p+), (R2p+), (R5p+), (RTp+), (Mor01u), (Mor02u), (Mor03u), (Mor04u), (Mor05u), (Mor06u), (Mor07u), (Mor08u), (Mor09u), (Mor10u), (Mor11u), (Mor12u), (Mor13u), (Mor14u), (Mor15u), (Mor16u), (Mor17u), (Mor18u), (Mor19u), (Mor20u), (Mor21u), (Mor22u), (Mor23u), (Mor24u), (Mor25u), (Mor26u), (Mor27u), (Mor28u), (Mor29u), (Mor30u), (Mor31u), (Mor32u), (Mor01m), (Mor02 m), (Mor03m), (Mor04m), (Mor05m), (Mor06m), (Mor07m), (Mor08m), (Mor09m), (Mor10m), (Mor11m), (Mor12m), (Mor13m), (Mor14m), (Mor15m), (Mor16m), (Mor17m), (Mor18m), (Mor19m), (Mor20m). (Mor21m), (Mor22m ), (Mor23m), (Mor24m), (Mor25m), (Mor26m), (Mor27m), (Mor28m), (Mor29m), (Mor30m), (Mor31m), (Mor32m), (Mor01v), (Mor02v), (Mor03v), (Mor04v), (Mor05v), (Mor06v), (Mor07v), (Mor08v), (Mor09v), (Mor10v), (Mor11v), (Mor12v), (Mor13v), (Mor14v), (Mor15v), (Mor16v), (Mor17v), (Mor18v), (Mor19v), (Mor20v), (Mor21v), (Mor22v), (Mor23v), (Mor24v), (Mor25v), (Mor26v), (Mor27v), (Mor28v), (Mor29v), (Mor30v), (Mor31v), (Mor32v), (Mor01e), (Mor02e), (Mor03e), (Mor04e), (Mor05e), (Mor06e), (Mor07e), (Mor08e), (Mor09e), (Mor10e), (Mor11e), (Mor12e), (Mor13e), (Mor14e), (Mor15e), (Mor16e), (Mor17e), (Mor18e), (Mor19e), (Mor20e), (Mor21e), (Mor22e), (Mor23e), (Mor24e), (Mor25e), (Mor26e), (Mor27e), (Mor28e), (Mor29e), (Mor30e), (Mor31e), (Mor32e), (Mor01p), (Mor02p), (Mor03p), (Mor04p), (Mor05p), (Mor06p), (Mor07p), (Mor08p), (Mor09p), (Mor10p), (Mor11p), (Mor12p), (Mor13p), (Mor14p), (Mor15p), (Mor16p), (Mor17p), (Mor18p), (Mor19p), (Mor20p), (Mor21p), (Mor22p), (Mor23p), (Mor24p), (Mor25p), (Mor26p), (Mor27p), (Mor28p), (Mor29p), (Mor30p), (Mor31p), (Mor32p),(RDF010u), (RDF015u), (RDF020u), (RDF025u), (RDF030u), (RDF035u), (RDF040u), (RDF045u), (RDF050u), (RDF055u), (RDF060u), (RDF065u), (RDF070u), (RDF075u), (RDF080u), (RDF085u), (RDF090u), (RDF095u), (RDF100u), (RDF105u), (RDF110u), (RDF115u), (RDF120u), (RDF125u), (RDF130u), (RDF135u), (RDF140u), (RDF145u), (RDF150u), (RDF155u), (RDF010m), (RDF015m), (RDF020m), (RDF025m), (RDF030m), (RDF035m), (RDF040m), (RDF045m), (RDF050m), (RDF055m), (RDF060m), (RDF065m),(RDF070m), (RDF075m), (RDF080m), (RDF085m), (RDF090m), (RDF095m), (RDF100m), (RDF105m), (RDF110m), (RDF115m), (RDF120m), (RDF125m), (RDF130m), (RDF135m), (RDF140m), (RDF145m), (RDF150m), (RDF155m), (RDF010v), (RDF015v), (RDF020v), (RDF025v), (RDF030v), (RDF035v), (RDF040v), (RDF045v), (RDF050v), (RDF055v), (RDF060v), (RDF065v), (RDF070v), (RDF075v), (RDF080v), (RDF085v), (RDF090v), (RDF095v), (RDF100v), (RDF105v), (RDF110v), (RDF115v), (RDF120v), (RDF125v), (RDF130v), (RDF135v), (RDF140v), (RDF145v), (RDF150v), (RDF155v), (RDF010e), (RDF015e), (RDF020e), (RDF025e), (RDF030e), (RDF035e), (RDF040e), (RDF045e), (RDF050e), (RDF055e), (RDF060e), (RDF065e), (RDF070e), (RDF075e), (RDF080e), (RDF085e), (RDF090e), (RDF095e), (RDF100e), (RDF105e), (RDF110e), (RDF115e), (RDF120e), (RDF125e), (RDF130e), (RDF135e), (RDF140e), (RDF145e), (RDF150e), (RDF155e), (RDF010p), (RDF015p), (RDF020p), (RDF025p), (RDF030p), (RDF035p), (RDF040p), (RDF045p), (RDF050p), (RDF055p), (RDF060p), (RDF065p), (RDF070p), (RDF075p), (RDF080p), (RDF085p), (RDF090p), (RDF095p), (RDF100p), (RDF105p), (RDF110p), (RDF115p), (RDF120p), (RDF125p), (RDF130p), (RDF135p), (RDF140p), (RDF145p), (RDF150p), (RDF155p), (L1u), (L2u), (L3u), (P1u), (P2u), (G1u), (G2u), (G3u), (E1u), (E2u), (E3u), (L1m), (L2m), (L3m), (P1m), (P2m), (G1m), (G2m), (G3m), (E1m), (E2m), (E3m), (L1v), (L2v), (L3v), (P1v), (P2v), (G1v), (G2v), (G3v), (E1v), (E2v), (E3v), (L1e), (L2e), (L3e), (P1e), (P2e), (G1e), (G2e), (G3e), (E1e), (E2e), (E3e), (L1p), (L2p), (L3p), (P1p), (P2p), (G1p), (G2p), (G3p), (E1p), (E2p), (E3p), (L1s), (L2s), (L3s), (P1s), (P2s), (G1s), (G2s), (G3s), (E1s), (E2s), (E3s), (Tu), (Tm), (Tv), (Te), (Tp), (Ts), (Au), (Am), (Av), (Ae), (Ap), (As), (Gu), (Gm), (Gs), (Ku), (Km), (Kv), (Ke), (Kp), (Ks), (Du), (Dm), (Dv), (De), (Dp), (Ds), (Vu), (Vm), (Vv), (Ve), (Vp), (Vs). |
| Topological | First Zagreb index (ZM1), first Zagreb index by valence vertex degrees (ZM1V), second Zagreb index (ZM2), Zagreb index by valence vertex degrees (ZM2V), quadratic index (Qindex), Narumi simple topological index (logfunction) (SNar), Narumi harmonic topological index (HNar), Narumi geometric topological index (GNar), total structureconnectivity index (Xt), Pogliani index (Dz), Pogliani index (Ram), polarity number (Pol), log of product of row sums (LPRS), (VDA), mean square distance index (MSD), Schultz Molecular TopologicalIndex(SMTI), Schultz Molecular Topological Index by valence vertex degrees (SMTIV), Gutman Molecular TopologicalIndex(GMTI), Gutman Molecular Topological Index by valence vertex degrees (GMTIV), Xu index (Xu), superpendenticindex(SPI), (W), (WA), (Har), (Har2), quasi-Wiener index (Kirchhoff number) from Laplace matrix (QW), first Mohar index fromLaplace matrix (TI1), second Mohar index from Laplace matrix (TI2), spanning tree number (log function) from Laplacematrix(STN), (HyDp), (RHyDp), Wiener-like index from topological distance matrix (w), (ww), (Rww), Wiener-like index fromdistance/detour matrix (D**/**D), all-path Wiener index (Wap), (WhetZ), (Whetm), (Whetv), (Whete), (Whetp), (J), (JhetZ), (Jhetm), (Jhetv), (Jhete), (Jhetp), maximal electrotopological negative variation (MAXDN), maximal electrotopological positive variation (MAXDP), molecular electrotopological variation (DELS), E-state topological parameter (TIE), Kier symmetry index (S0K), 1-path Kier alpha-modified shape index (S1K), 2-pathKier alpha-modified shape index (S2K), 3-path Kier alpha-modified shapeindex(S3K), Kier flexibility index (PHI), Kier benzene-likeliness index (BLI), path/walk 2 - Randic shape index( (PW2),path/walk 3 - Randic shape index (PW3), path/walk 4 - Randic shape index (PW4), path/walk 5 - Randic shape index (PW5),2D Petitjean shape index (PJI2), eccentric connectivity index (CSI), eccentricity (ECC), average eccentricity (AECC), eccentric (DECC), mean distance degree deviation (MDDD), unipolarity(UNIP), centralization (CENT), variation (VAR), Balaban centric index (BAC), lopping (Lop), radial centric information index (ICR), (D**/**Dr05), (D**/**Dr06), (D**/**Dr10), sum of topological distances between N..N (T (N..N)), sum oftopological distances betweenN..O (T (N..O)), sum of topological distances between N..Cl (T (N..Cl)), sum of topologicaldistancesbetweenN..Br (T (N..Br)), sum of topologicaldistances between O..O (T (O..O)), sum of topologicaldistancesbetweenO..S (T (O..S)), sum of topological distances between O..F (T (O..F)), sum of topologicaldistancesbetweenO..Cl (T (O..Cl)), sum of topologicaldistances between O..Br (T (O..Br)), sum of topological distances between S..Cl (T (S..Cl)), sum of topological distances between F..Cl (T (F..Cl)), sum of topological distances between Cl..Cl (T (Cl..Cl)), sum of topological distances between Cl..Br (T (Cl..Br)), molecular walk count of order 1(MWC01), molecular walk count of order 2 (MWC02), molecular walk count of order 3(MWC03), molecular walk count of order 4(MWC04), molecular walk count of order 5(MWC05), molecular walk count of order 6(MWC06),molecular walk count of order 7 (MWC07), molecular walk count of order 8(MWC08), molecular walk count of order 9 (MWC09),molecular walk count of order 10 (MWC10), total walk count(TWC), self-returning walk count of order 1(SRW01),self-returning walk count of order 2 (SRW02), self-returning walk count of order 4(SRW04), self-returning walk count of order 5(SRW05), self-returning walk count of order 6(SRW06),self-returning walk count of order 7 (SRW07), self-returning walk count of order 8(SRW08),self-returning walk count of order 9 (SRW09), self-returning walk count of order 10(SRW10), molecular path count of order 1 (Gordon-Scantlebury index) (MPC01), molecular path count of order 2 (MPC02), molecular path count of order 3 (MPC03), molecular path count of order 4 (MPC04), molecular path count of order 5 (MPC05), molecular path count of order 6 (MPC06), molecular path count of order 7 (MPC07), molecular path count of order 8 (MPC08), molecular path count of order 9 (MPC09), molecular path count of order 10 (MPC10), molecular multiple path count oforder 1 (piPC01), molecular multiple path count of order 2(piPC02), molecular multiple path count of order 3(piPC03), molecular multiple path count of order 4(piPC04), molecular multiple path count of order 5(piPC05), molecular multiple path count of order 6(piPC06), molecular multiple path count of order 7(piPC07), molecular multiple path count of order 8(piPC08), molecular multiple path count of order 9(piPC09), molecular multiple path count oforder 10(piPC10), total path count (TPC), conventional bond order (piID), ratio of multiple pathcount over path count(PCR), difference between multiple path count and path count (PCD), Randic ID number (CID), Balaban ID number (BID), connectivity index of order 0 (X0), connectivity index of order 1(X1), connectivity index of order 2(X2), connectivity index of order 3(X3), connectivity index of order 4(X4), connectivity index of order 5(X5), average connectivity index of order 0 (X0A), average connectivity index of order 1 (X1A), average connectivity index of order 2 (X2A), average connectivity index of order 3 (X3A), valence connectivity index of order0 (X0v), valence connectivity index of order 1(X1v), valence connectivity index of order 2(X2v), valence connectivity index of order 3(X3v), valence connectivity index of order 4(X4v), valence connectivity index of order 5(X5v), averagevalence connectivity index of order 0 (X0Av), averagevalence connectivity index of order 1(X1Av), averagevalence connectivity index of order 2(X2Av), averagevalence connectivity index of order 3(X3Av), averagevalence connectivity index of order 4(X4Av), averagevalence connectivity index of order 5(X5Av), solvation connectivity index of order 0 (X0sol), solvation connectivity index of order 1(X1sol),solvation connectivity index of order 2 (X2sol), solvation connectivity index of order 3 (X3sol), solvation connectivity index of order 4(X4sol), solvation connectivity index of order 5(X5sol), modified Randic index (XMOD), reciprocaldistance sum Randic-like index (RDCHI), reciprocal distance sum inverse Randic-like index (RDSQ), (ATS1m), (ATS2m), (ATS3m), (ATS4m), (ATS5m), (ATS6m), (ATS7m), (ATS8m), (ATS1v), (ATS2v), (ATS3v), (ATS4v), (ATS5v), (ATS6v), (ATS7v), (ATS8v), (ATS1e), (ATS2e), (ATS3e), (ATS4e), (ATS5e), (ATS6e), (ATS7e), (ATS8e), (ATS1p), (ATS2p), (ATS3p), (ATS4p), (ATS5p), (ATS6p), (ATS7p), (ATS8p), Moran autocorrelation of lag 1 weighted by mass (MATS1m), Moran autocorrelation of lag 2 weighted by mass (MATS2m), Moran autocorrelation of lag 3 weighted by mass (MATS3m), Moran autocorrelation of lag 4 weighted by mass (MATS4m), Moran autocorrelation of lag 5 weighted by mass (MATS5m), Moran autocorrelation of lag 6 weighted by mass (MATS6m), Moran autocorrelation of lag 7 weighted by mass (MATS7m), Moran autocorrelation of lag 8 weighted by mass (MATS8m), (MATS1v), (MATS2v), (MATS3v), (MATS4v), (MATS5v), (MATS6v), (MATS7v), (MATS8v), Moran autocorrelation - lag 1 / weighted by atomic Sanderson electronegativities (MATS1e),Moran autocorrelation - lag 2 / weighted by atomic Sanderson electronegativities (MATS2e), Moran autocorrelation - lag 3 / weighted by atomic Sanderson electronegativities (MATS3e), Moran autocorrelation - lag 4 / weighted by atomic Sanderson electronegativities (MATS4e), Moran autocorrelation - lag 5 / weighted by atomic Sanderson electronegativities (MATS5e), Moran autocorrelation - lag 6 / weighted by atomic Sanderson electronegativities (MATS6e), Moran autocorrelation - lag 7 / weighted by atomic Sanderson electronegativities (MATS7e), Moran autocorrelation - lag 8 / weighted by atomic Sanderson electronegativities (MATS8e), Moranautocorrelation - lag 1 / weighted by atomic polarizabilities (MATS1p), Moran autocorrelation - lag 2 / weighted by atomic polarizabilities(MATS2p), Moran autocorrelation - lag 3 / weighted by atomic polarizabilities(MATS3p), Moranautocorrelation - lag 4 / weighted by atomic polarizabilities(MATS4p),Moran autocorrelation - lag 5 / weighted by atomic polarizabilities (MATS5p), Moran autocorrelation - lag 6 / weighted by atomic polarizabilities (MATS6p), Moran autocorrelation - lag 7 / weighted by atomic polarizabilities(MATS7p), Moran autocorrelation - lag 8 / weighted by atomic polarizabilities(MATS8p), (GATS1m), (GATS2m), (GATS3m), (GATS4m), (GATS5m), (GATS6m), (GATS7m), (GATS8m), (GATS1v), (GATS2v), (GATS3v), (GATS4v), (GATS5v), (GATS6v), (GATS7v), (GATS8v), (GATS1e), (GATS2e), (GATS3e), (GATS4e), (GATS5e), (GATS6e), (GATS7e), (GATS8e), (GATS1p), (GATS2p), (GATS3p), (GATS4p), (GATS5p), (GATS6p), (GATS7p), (GATS8p), information index onmolecular size (ISIZ), x total information index on atomic composition (IAC), (AAC), mean information content on the distanceequalit(IDE), x mean information content on the distance magnitude (IDM), mean information content on the distance degree equality (IDDE), mean information content on the distancedegree magnitude (IDDM), total information content on the distance equality (IDET), total information content on thedistance magnitude (IDMT), mean information content on the vertex degree equality (IVDE), mean information content onthe vertex degree magnitude (IVDM), graph vertex complexity index (HVcpx), graph distance complexity index (logfunction) (HDcpx), Balaban U index (Uindex), Balaban V index (Vindex), Balaban X index (Xindex), BalabanYindex(Yindex), Information Content index (neighborhood symmetry of 0-order) (IC0), Total Information Content index(neighborhood symmetry of 0-order) (TIC0), Structural Information Content index (neighborhood symmetry of 0-order) (SIC0), Complementary Information Content index (neighborhood symmetry of 0-order) (CIC0), Bond InformationContent index (neighborhood symmetry of 0-order) (BIC0), Information Content index (neighborhood symmetry of 1-order) (IC1), Total Information Content index (neighborhood symmetry of 1-order) (TIC1), Structural InformationContent index (neighborhood symmetry of 1-order) (SIC1), Complementary Information Content index (neighborhoodsymmetry of 1-order) (CIC1), Bond Information Content index (neighborhood symmetry of 1-order) (BIC1), InformationContent index (neighborhood symmetry of 2-order) (IC2), Total Information Content index (neighborhood symmetry of 2-order) (TIC2), Structural Information Content index (neighborhood symmetry of 2-order)(SIC2), ComplementaryInformation Content index (neighborhood symmetry of 2-order) (CIC2), Bond Information Content index (neighborhoodsymmetry of 2-order) (BIC2), Information Content index (neighborhood symmetry of 3-order) (IC3), Total InformationContent index (neighborhood symmetry of 3-order) (TIC3), Structural Information Content index (neighborhoodsymmetry of 3-order) (SIC3), Complementary Information Content index (neighborhood symmetry of 3-order) (CIC3), Bond Information Content index (neighborhood symmetry of 3-order) (BIC3), Information Content index (neighborhoodsymmetry of 4-order) (IC4), Total Information Content index (neighborhood symmetry of 4-order) (TIC4), StructuralInformation Content index (neighborhood symmetry of 4-order) (SIC4), Complementary Information Content index(neighborhood symmetry of 4-order) (CIC4), Bond Information Content index (neighborhood symmetry of 4-order) (BIC4), Information Content index (neighborhood symmetry of 5-order) (IC5), Total Information Content index(neighborhood symmetry of 5-order) (TIC5), Structural Information Content index (neighborhood symmetry of 5-order) (SIC5), Complementary Information Content index (neighborhood symmetry of 5-order) (CIC5), Bond InformationContent index (neighborhood symmetry of 5-order) (BIC5), (EPS0), (EPS1), Eigenvalue 02 from edge adj. matrix weighted by edge degrees (EEig02x), Eigenvalue 03 from edge adj. matrix weighted by edge degrees (EEig03x), Eigenvalue 04 from edge adj. matrix weighted by edge degrees (EEig04x), Eigenvalue 05 from edge adj. matrix weighted by edge degrees (EEig05x), Eigenvalue 06 from edge adj. matrix weighted by edge degrees (EEig06x), Eigenvalue 07 from edge adj. matrix weighted by edge degrees (EEig07x), Eigenvalue 08 from edge adj. matrix weighted by edge degrees (EEig08x), Eigenvalue 09 from edge adj. matrix weighted by edge degrees (EEig09x), Eigenvalue 10 from edge adj. matrix weighted by edge degrees (EEig10x), Eigenvalue 11 from edge adj. matrix weighted by edge degrees (EEig11x), Eigenvalue 12 from edge adj. matrix weighted by edge degrees (EEig12x),Eigenvalue 13 from edge adj. matrix weighted by edge degrees (EEig13x), Eigenvalue 14 from edge adj. matrix weighted by edge degrees (EEig14x), Eigenvalue 15 from edge adj. matrix weighted by edge degrees (EEig15x), Autocorrelation. Eigenvalue 01 from edge adj. matrixweighted by dipole moments (EEig01d), Eigenvalue 02 from edge adj. matrixweighted by dipole moments (EEig02d), Eigenvalue 03 from edge adj. matrixweighted by dipole moments (EEig03d), Eigenvalue 04 from edge adj. matrixweighted by dipole moments (EEig04d), Eigenvalue 05 from edge adj. matrixweighted by dipole moments (EEig05d), Eigenvalue 06 from edge adj. matrixweighted by dipole moments (EEig06d), Eigenvalue 07 from edge adj. matrixweighted by dipole moments (EEig07d), Eigenvalue 08 from edge adj. matrixweighted by dipole moments (EEig08d), Eigenvalue 09 from edge adj. matrixweighted by dipole moments (EEig09d), Eigenvalue 10 from edge adj. matrixweighted by dipole moments (EEig10d), Eigenvalue 11 from edge adj. matrixweighted by dipole moments (EEig11d), Eigenvalue 12 from edge adj. matrixweighted by dipole moments (EEig12d), Eigenvalue 13 from edge adj. matrixweighted by dipole moments (EEig13d), Eigenvalue 14 from edge adj. matrixweighted by dipole moments (EEig14d), Eigenvalue 15 from edge adj. matrixweighted by dipole moments (EEig15d), (EEig01r), (EEig02r), (EEig03r), (EEig04r), (EEig05r), (EEig06r), (EEig07r), (EEig08r), (EEig09r), (EEig10r), (EEig11r), (EEig12r), (EEig13r), (EEif14r), (EEig15r), (ESpm02u), (ESpm03u), (ESpm04u), (ESpm05u), )ESpm06u), (ESpm07u), (ESpm08u), (ESpm09u), (ESpm10u), (ESpm11u), (ESpm12u), (ESpm13u), (ESpm14u), (ESpm15u), (ESpm01x), (ESpm02x), (ESpm03x), (ESpm04x), (ESpm05x), (ESpm06x), (ESpm07x), (ESpm08x), (ESpm09x), (ESpm10x), (ESpm11x), (ESpm12x), (ESpm13x), (ESpm14x), (ESpm15x), (ESpm01d), (ESpm02d), (ESpm03d), (ESpm04d), (ESpm05d), (ESpm06d), (ESpm07d), (ESpm08d), (ESpm09d), (ESpm10d), (ESpm11d), (ESpm12d), (ESpm13d), (ESpm14d), (ESpm15d), (ESpm01r), (ESpm02r), (ESpm03r), (ESpm04r), (ESpm05r), (ESpm06r), (ESpm07r), (ESpm08r), (ESpm09r), (ESpm10r), (ESpm11r), (ESpm12r), (ESpm13r), (ESpm14r), (ESpm15r), (BEHm1), (BEHm2), (BEHm3), (BEHm4), (BEHm5), (BEHm6), (BEHm7), (BEHm8), (BELm2), (BELm3), (BELm4), (BELm5), (BELm6), (BELm7), (BELm8), (BEHv2), (BEHv3), (BEHv4), (BEHv5), (BEHv6), (BEHv7), (BEHv8), (BELv2), (BELv3), (BELv4), (BELv5), (BELv6), (BELv7), (BELv8), (BEHe2), (BEHe3), (BEHe4), (BEHe5), (BEHe6), (BEHe7), (BEHe8), (BELe2), (BELe3), (BELe4), (BELe5), (BELe6), (BELe7), (BELe8), (BEHp2), (BEHp3), (BEHp4), (BEHp5), (BEHp6), (BEHp7), (BEHp8), (BELp2), (BELp3), (BELp4), (BELp5), (BELp6), (BELp7), (BELp8), (GGI1), (GGI2), (GGI3), (GGI4), (GGI5), (GGI6), (GGI7), (GGI8), (GGI9), (GGI10), (JGI1), (JGI2), (JGI3), (JGI4), (JGT), (Eig1Z), (Eig1m), (Eig1v), (Eig1e), (Eig1p), (SEigZ), (SEigm), (SEigv), (SEige), (SEigp), (AEigZ), (AEigm), (AEigv), (AEige), (AEigp), (VEA1), (VEA2), (VRA1), (VRA2), (VED1), (VED2), (VRD1), (VRD2), (VEZ1), (VEZ2), (VRZ1), (VRZ2), (VEm1), (VEm2), (VRm1), (VRm2), (VEv1), (VEv2), (VRv1), (VRv2), (VEe1), (VEe2), (VRe1), (VRe2), (VEp1), (VEp2), (VRp1), (VRp2), (DP01), (DP02), (DP03), (DP04), (DP05), (DP06), (DP07), (DP08), (DP09), (DP10), (DP11), (DP12), (DP13), (DP14), (DP15), (DP16), (DP17), (DP18), (DP19), (DP20), shape profile no.01(SP01), shape profile no.02(SP02), shape profile no.03(SP03), shape profile no.04(SP04), shape profile no.05 (SP05), shape profile no.06(SP06), shape profile no.07(SP07), shape profile no.08(SP08), shape profile no.09(SP09), shape profile no.10(SP10), shape profile no.11(SP11), shape profile no.12(SP12), shape profile no.13(SP13), shape profile no.14(SP14), shape profile no.15(SP15), shape profile no.16(SP16), shape profile no.17(SP17), shape profile no.18(SP18), shape profile no.19(SP19), shape profile no.20(SP20), (SHP2). |
| Constitutional  and  Molecular Property | Molecular weight (MW), average molecular weight (AMW), sum of atomic van der Waals volumes (scaled on Carbonatom) (Sv), sum of atomic Sanderson electronegativities (scaled on Carbon atom) (Se), sum of atomic polarizabilities(scaled on Carbon atom) (Sp), (Ss), mean atomic van der Waalsvolume (scaled on Carbon atom (Mv), mean atomic Sanderson electronegativity (scaled on Carbon atom) (Me), meanatomicpolarizability (scaled on Carbon atom) (Mp), (Ms), numberof atoms (nAT), number of non-H atoms (nSK), number of bonds (nBT), number of non-H bonds (nBO), number of multiple bonds (nBM), sum of conventional bond orders (H-depleted) (SCBO), (ARR), (nCIC), (nCIR), number of rotatable bonds (RBN), rotatable bond fraction (RBF), number of double bonds (nDB), number of triple bonds (nTB), number of aromatic bonds (nAB), number ofHydrogen atoms (nH), number of Carbon atom (nC), number of Nitrogen atoms (nN), number of Oxygen atoms (nO), number of Sulfur atoms (nS), number of Fluorine atoms (nF), number ofChlorine atoms (nCL), number of Bromine atoms (nBR), number of halogen atoms (nX), (nR05), (nR06), (nR09), (nR10), (nBnz), (Ui), (Hy), (AMR), TPSA(NO), TPSA(Tot), (MLOGP), (MLOGP2), (ALOGP), (ALOGP2). |
| Functional Group  and  Atom-Centered Fragments | number of terminal primary C(sp3) (nCp), number of total secondary C(sp3) (nCs), number of total tertiary C(sp3) (nCt), number of ring secondary C(sp3) (nCrs), number of ring tertiary C(sp3) (nCrt), number of aromatic C(sp2) (nCar), number of unsubstituted benzene C(sp2) (nCbh), - number of substituted benzene C(sp2) (nCb-), number of non-aromaticconjugated C(sp2) (nCconj), (nRCOOR), number of esters (aromatic) (nArCOOR), (nRCONHR), (nArCHO), (nArNH2), (nRNHR), (nArNHR), (nRNR2), (nArNR2), (nArCN), number of positively chargedN(nN**+**), number of nitro groups (aromatic) (nArNO2), number of hydroxyl group (nROH), (nArOH), (nOHp), (nOHs), number of ethers (aliphatic) (nROR), number of ethers (aromatic) (nArOR), (nRSR), number of CH2RX (nCH2RX), (nCRX3), number of X on aromatic ring (nArX), (nPyrrolidines), (nPyrazoles), (nPyridines), number of donor atoms for H-bonds (N and O) (nHDon), number of acceptor atoms for H-bonds (N,O,F) (nHAcc), (nHBonds), CH3R/ CH (C-001), CH2R2 (C-002), CHR3 (C-003), CH3X (C-005), CH2RX(v) (C-006), (C-007), CHR2X (C-008), (C-011), (C-013), R--CH—R (C-024), R--CR—R (C-025), R--CX—R (C-026), (C-027), (C-028), (C-029), (C-033), (C-037), R-C(=X)-X / R-C#X /X=C=X (C-040), H attached to C0(sp3) no X attached to next C (H-046), H attached to C1(sp3)/C0(sp2) (H-047), H attached to C2(sp3)/C1(sp2)/C0(sp) (H-048), (H-049), H attached to heteroatom (H-050), H attached to alpha-C (H-051), H attachedto C0(sp3) with 1X attached to next C (H-052), (H-053), alcohol (O-056), (O-057), (O-058), (O-059), Al-O-Ar / Ar-O-Ar / R..O..R / R-OC=X (O-060), O— (O-061), (N-067), (N-068), (N-069), (N-070), (N-071), (N-072), (N-073), (N-074), (N-075), (N-076), (F-083), Cl attached to C1(sp2) (Cl-089), (Br-091), (S-107). |

^†^ Part of this descriptor list has been published in reference (Batra et al. 2015).
